# Tooth Powders and Tooth Washes

**Published:** 1890-11

**Authors:** 


### TOOTH POWDERS AND TOOTH WASHES.

Powders and washes for the teeth should be used with great care. Regarding them, especially, the well worn but pertinent caution to beware of strolling vendors applies with the deepest import. Every one has a desire for white and beautiful teeth, and the itinerant who boasts loudly of the power of his preparations to "whiten the blackest teeth to look like ivory in one minute !" catches the popular ear and sympathy on the spot. There is nothing remarkable in the fact that what he claims can be demonstrated. Any chemist or apothecary can concoct a preparation which will do all this—and more. If used but a short time it will destroy the enamel, and with it, of course, the entire set of teeth ; since the phenomenal result is and can be reached only by the destruction of a small portion of the outer surface of the enamel. The result is the same whether the agent be wash or powder, since the latter simply contains the chemicals of the former in an undissolved form. All strong acids or alkalies should be avoided in the mouth, and if there is a doubt as to the composition of any preparation in this respect, let it be tested with a bit of litmus paper. This paper can be obtained at any drug store, and is in two colors—blue and red. The blue, if dampened with an acid solution, will turn red, and the rapidity and intensity of the change will indicate the acidity of the solution. The red indicates alkali by changing to blue, in the same manner.

Tooth powders, as a rule, should be soluble and slightly antacid. There is a class of insoluble powders which are of the most dangerous nature, of which powdered charcoal is a notable example. These consist of fine sharp particles, which being pressed by the brush between the teeth and gums or lodging between the teeth, may cause the most serious results, even to the destruction of the gums or the cement. The use of the brush in connection with powders, washes or other treatment of the teeth should be gentle. Bleeding of the gums is always a danger signal. It shows that the skin has been broken, inviting the absorption into the system of any poisonous or foreign matters which may be present in the mouth. If the gums are very tender a soft brush should be used, and used very gently, till they have hardened sufficiently to withstand more vigorous treatment. Even then, the liability will be to err on the side of harshness.—*Health and Home.*

---

WATCHES AND NATURE.—A facetious watchmaker makes this unique comparison in the *Jeweler's Weekly* :—" A watch is like the human body. It is just as sensitive as the most delicate child and needs more care and protection than it ever receives.

" It is affected by climatic influences, and its vitals are just as liable to derangement as those of our bodies. Its heart beats govern its action and its hands and face tell its condition at all times.

"If I were to classify the diseases of watches, I should say that the one where the works are clogged with dirt and the oil has become stiff is analogous to our biliousness. This is the most common complaint watch doctors find, and unless the owner of the watch makes it a rule to submit it to a reputable repairer he will probably be victimized, just as human patients are when they consult quack doctors."

